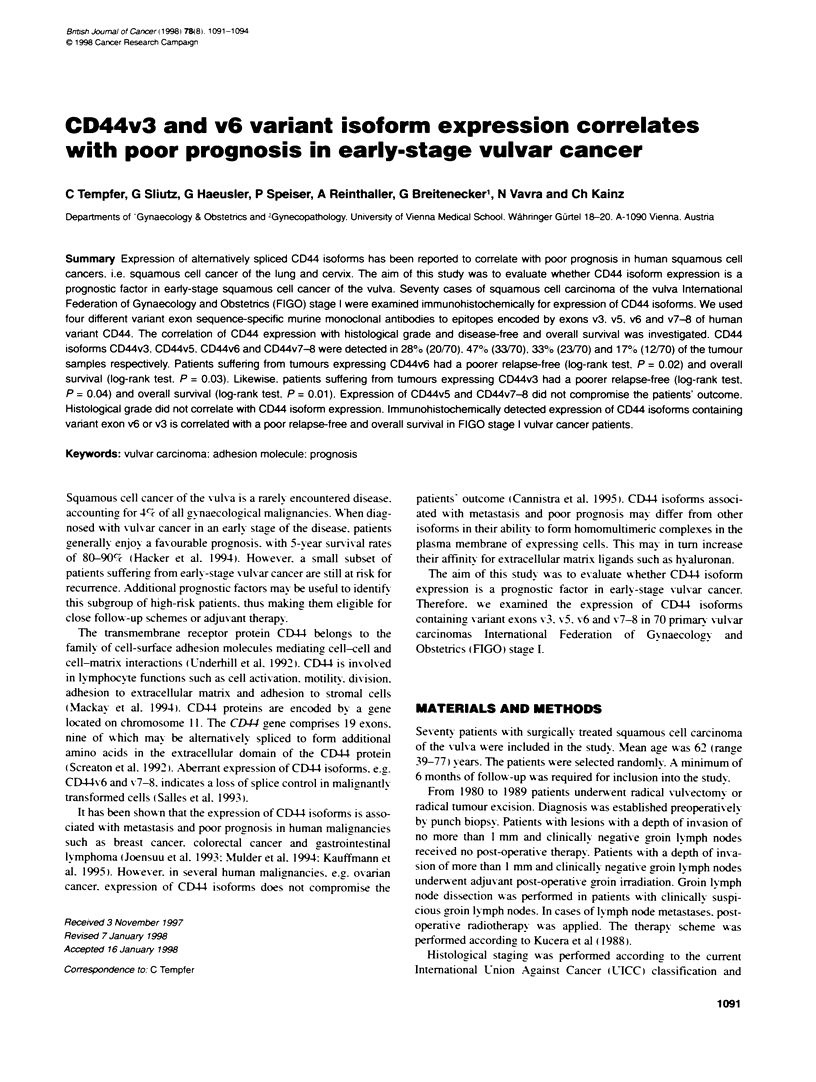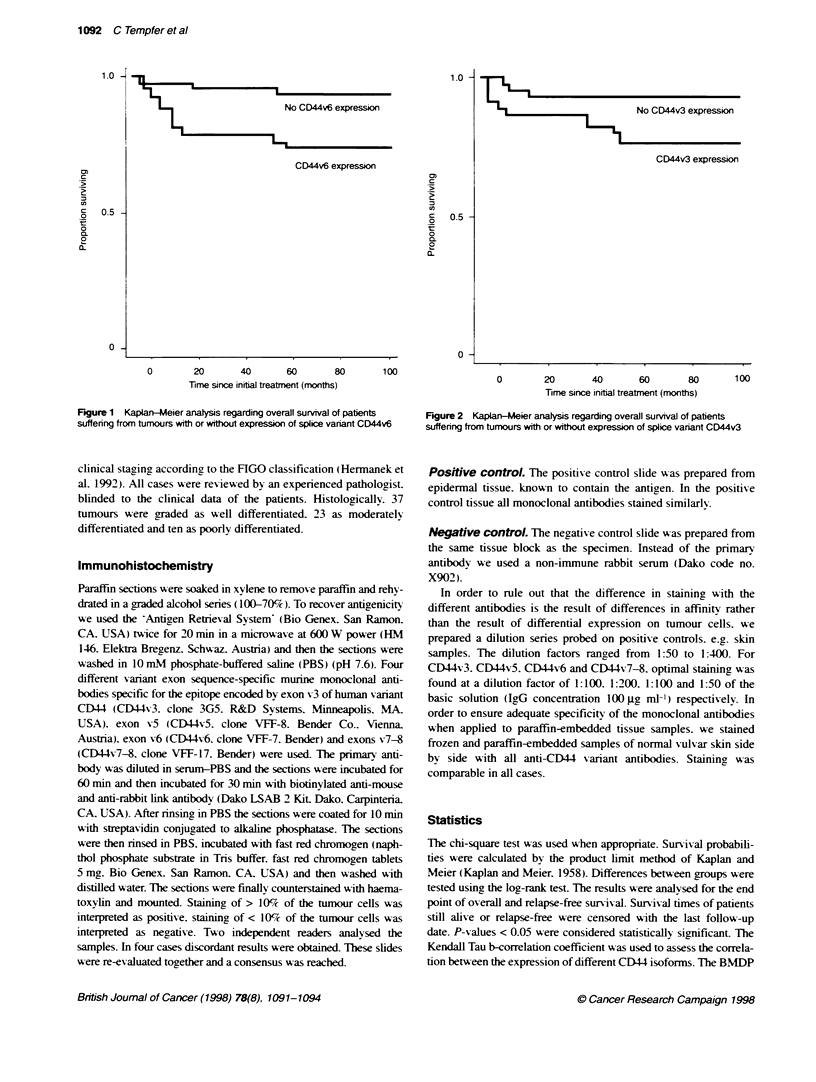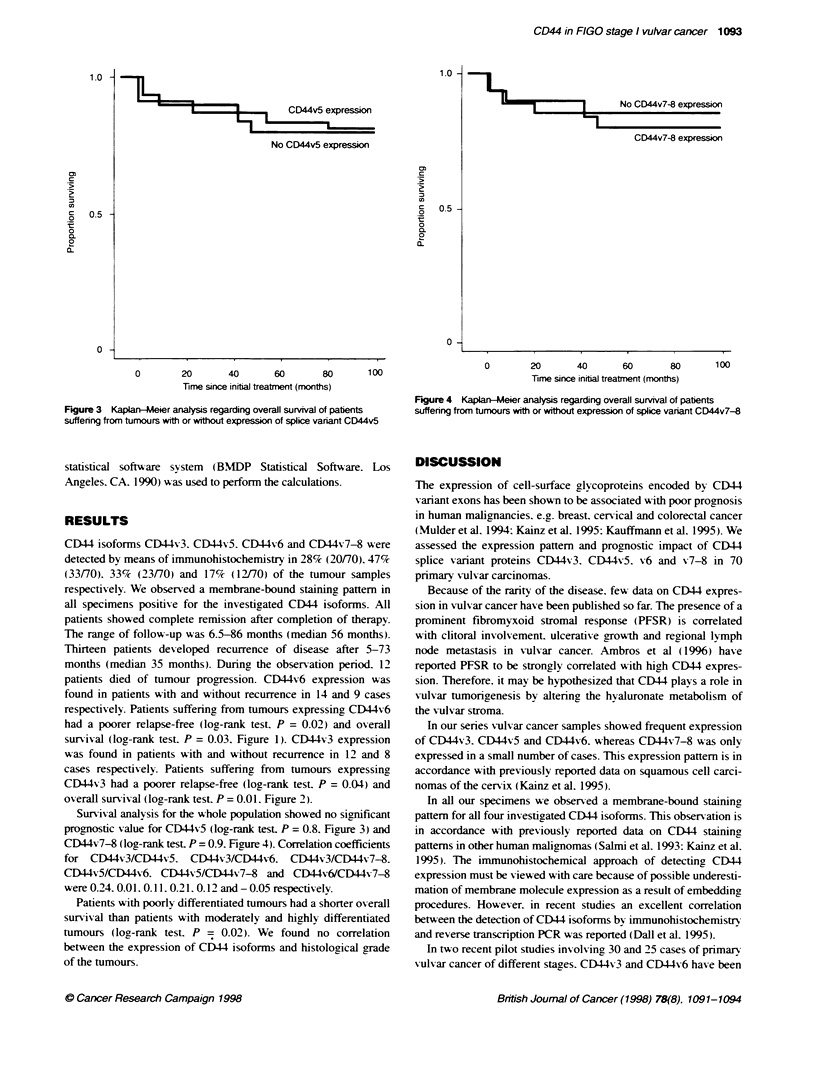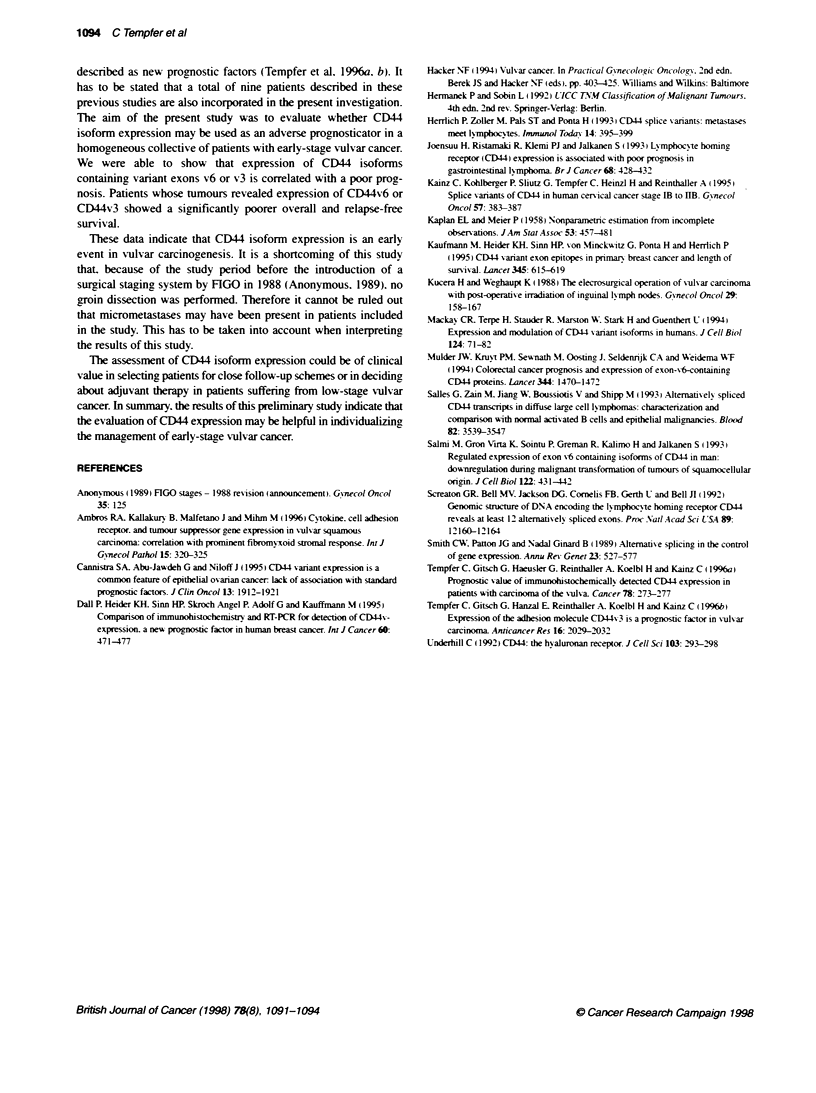# CD44v3 and v6 variant isoform expression correlates with poor prognosis in early-stage vulvar cancer.

**DOI:** 10.1038/bjc.1998.633

**Published:** 1998-10

**Authors:** C. Tempfer, G. Sliutz, G. Haeusler, P. Speiser, A. Reinthaller, G. Breitenecker, N. Vavra, C. Kainz

**Affiliations:** Department of Gynaecology & Obstetrics, University of Vienna Medical School, Austria.

## Abstract

Expression of alternatively spliced CD44 isoforms has been reported to correlate with poor prognosis in human squamous cell cancers, i.e. squamous cell cancer of the lung and cervix. The aim of this study was to evaluate whether CD44 isoform expression is a prognostic factor in early-stage squamous cell cancer of the vulva. Seventy cases of squamous cell carcinoma of the vulva International Federation of Gynaecology and Obstetrics (FIGO) stage I were examined immunohistochemically for expression of CD44 isoforms. We used four different variant exon sequence-specific murine monoclonal antibodies to epitopes encoded by exons v3, v5, v6 and v7-8 of human variant CD44. The correlation of CD44 expression with histological grade and disease-free and overall survival was investigated. CD44 isoforms CD44v3, CD44v5, CD44v6 and CD44v7-8 were detected in 28% (20/70), 47% (33/70), 33% (23/70) and 17% (12/70) of the tumour samples respectively. Patients suffering from tumours expressing CD44v6 had a poorer relapse-free (log-rank test, P = 0.02) and overall survival (log-rank test, P = 0.03). Likewise, patients suffering from tumours expressing CD44v3 had a poorer relapse-free (log-rank test, P = 0.04) and overall survival (log-rank test, P = 0.01). Expression of CD44v5 and CD44v7-8 did not compromise the patients' outcome. Histological grade did not correlate with CD44 isoform expression. Immunohistochemically detected expression of CD44 isoforms containing variant exon v6 or v3 is correlated with a poor relapse-free and overall survival in FIGO stage I vulvar cancer patients.


British Journal of Cancer (1998) 78(8), 1091–1094
© 1998 Cancer Research Campaign

### CD44v3 and v6 variant isoform expression correlates with poor prognosis in early-stage vulvar cancer

**C Tempfer, G Sliutz, G Haeusler, P Speiser, A Reinthaller, G Breitenecker[1], N Vavra and Ch Kainz**

Departments of [1]Gynaecology & Obstetrics and [2]Gynecopathology, University of Vienna Medical School, Währinger Gürtel 18–20, A-1090 Vienna, Austria

**Summary** Expression of alternatively spliced CD44 isoforms has been reported to correlate with poor prognosis in human squamous cell cancers, i.e. squamous cell cancer of the lung and cervix. The aim of this study was to evaluate whether CD44 isoform expression is a prognostic factor in early-stage squamous cell cancer of the vulva. Seventy cases of squamous cell carcinoma of the vulva International Federation of Gynaecology and Obstetrics (FIGO) stage I were examined immunohistochemically for expression of CD44 isoforms. We used four different variant exon sequence-specific murine monoclonal antibodies to epitopes encoded by exons v3, v5, v6 and v7–8 of human variant CD44. The correlation of CD44 expression with histological grade and disease-free and overall survival was investigated. CD44 isoforms CD44v3, CD44v5, CD44v6 and CD44v7–8 were detected in 28% (20/70), 47% (33/70), 33% (23/70) and 17% (12/70) of the tumour samples respectively. Patients suffering from tumours expressing CD44v6 had a poorer relapse-free (log-rank test, $P = 0.02$) and overall survival (log-rank test, $P = 0.03$). Likewise, patients suffering from tumours expressing CD44v3 had a poorer relapse-free (log-rank test, $P = 0.04$) and overall survival (log-rank test, $P = 0.01$). Expression of CD44v5 and CD44v7–8 did not compromise the patients' outcome. Histological grade did not correlate with CD44 isoform expression. Immunohistochemically detected expression of CD44 isoforms containing variant exon v6 or v3 is correlated with a poor relapse-free and overall survival in FIGO stage I vulvar cancer patients.

**Keywords:** vulvar carcinoma; adhesion molecule; prognosis

Squamous cell cancer of the vulva is a rarely encountered disease, accounting for 4% of all gynaecological malignancies. When diagnosed with vulvar cancer in an early stage of the disease, patients generally enjoy a favourable prognosis, with 5-year survival rates of 80–90% (Hacker et al, 1994). However, a small subset of patients suffering from early-stage vulvar cancer are still at risk for recurrence. Additional prognostic factors may be useful to identify this subgroup of high-risk patients, thus making them eligible for close follow-up schemes or adjuvant therapy.

The transmembrane receptor protein CD44 belongs to the family of cell-surface adhesion molecules mediating cell–cell and cell–matrix interactions (Underhill et al, 1992). CD44 is involved in lymphocyte functions such as cell activation, motility, division, adhesion to extracellular matrix and adhesion to stromal cells (Mackay et al, 1994). CD44 proteins are encoded by a gene located on chromosome 11. The *CD44* gene comprises 19 exons, nine of which may be alternatively spliced to form additional amino acids in the extracellular domain of the CD44 protein (Screaton et al, 1992). Aberrant expression of CD44 isoforms, e.g. CD44v6 and v7–8, indicates a loss of splice control in malignantly transformed cells (Salles et al, 1993).

It has been shown that the expression of CD44 isoforms is associated with metastasis and poor prognosis in human malignancies such as breast cancer, colorectal cancer and gastrointestinal lymphoma (Joensuu et al, 1993; Mulder et al, 1994; Kauffmann et al, 1995). However, in several human malignancies, e.g. ovarian cancer, expression of CD44 isoforms does not compromise the patients' outcome (Cannistra et al, 1995). CD44 isoforms associated with metastasis and poor prognosis may differ from other isoforms in their ability to form homomultimeric complexes in the plasma membrane of expressing cells. This may in turn increase their affinity for extracellular matrix ligands such as hyaluronan.

The aim of this study was to evaluate whether CD44 isoform expression is a prognostic factor in early-stage vulvar cancer. Therefore, we examined the expression of CD44 isoforms containing variant exons v3, v5, v6 and v7–8 in 70 primary vulvar carcinomas International Federation of Gynaecology and Obstetrics (FIGO) stage I.

### MATERIALS AND METHODS

Seventy patients with surgically treated squamous cell carcinoma of the vulva were included in the study. Mean age was 62 (range 39–77) years. The patients were selected randomly. A minimum of 6 months of follow-up was required for inclusion into the study.

From 1980 to 1989 patients underwent radical vulvectomy or radical tumour excision. Diagnosis was established preoperatively by punch biopsy. Patients with lesions with a depth of invasion of no more than 1 mm and clinically negative groin lymph nodes received no post-operative therapy. Patients with a depth of invasion of more than 1 mm and clinically negative groin lymph nodes underwent adjuvant post-operative groin irradiation. Groin lymph node dissection was performed in patients with clinically suspicious groin lymph nodes. In cases of lymph node metastases, post-operative radiotherapy was applied. The therapy scheme was performed according to Kucera et al (1988).

Histological staging was performed according to the current International Union Against Cancer (UICC) classification and

*Received 3 November 1997*
*Revised 7 January 1998*
*Accepted 16 January 1998*

*Correspondence to:* C Tempfer

**Figure 1** Kaplan–Meier analysis regarding overall survival of patients suffering from tumours with or without expression of splice variant CD44v6

**Figure 2** Kaplan–Meier analysis regarding overall survival of patients suffering from tumours with or without expression of splice variant CD44v3

clinical staging according to the FIGO classification (Hermanek et al. 1992). All cases were reviewed by an experienced pathologist, blinded to the clinical data of the patients. Histologically, 37 tumours were graded as well differentiated, 23 as moderately differentiated and ten as poorly differentiated.

### Immunohistochemistry

Paraffin sections were soaked in xylene to remove paraffin and rehydrated in a graded alcohol series (100–70%). To recover antigenicity we used the 'Antigen Retrieval System' (Bio Genex, San Ramon, CA, USA) twice for 20 min in a microwave at 600 W power (HM 146, Elektra Bregenz, Schwaz, Austria) and then the sections were washed in 10 mM phosphate-buffered saline (PBS) (pH 7.6). Four different variant exon sequence-specific murine monoclonal antibodies specific for the epitope encoded by exon v3 of human variant CD44 (CD44v3, clone 3G5, R&D Systems, Minneapolis, MA, USA), exon v5 (CD44v5, clone VFF-8, Bender Co., Vienna, Austria), exon v6 (CD44v6, clone VFF-7, Bender) and exons v7–8 (CD44v7–8, clone VFF-17, Bender) were used. The primary antibody was diluted in serum–PBS and the sections were incubated for 60 min and then incubated for 30 min with biotinylated anti-mouse and anti-rabbit link antibody (Dako LSAB 2 Kit, Dako, Carpinteria, CA, USA). After rinsing in PBS the sections were coated for 10 min with streptavidin conjugated to alkaline phosphatase. The sections were then rinsed in PBS, incubated with fast red chromogen (naphthol phosphate substrate in Tris buffer, fast red chromogen tablets 5 mg, Bio Genex, San Ramon, CA, USA) and then washed with distilled water. The sections were finally counterstained with haematoxylin and mounted. Staining of > 10% of the tumour cells was interpreted as positive, staining of < 10% of the tumour cells was interpreted as negative. Two independent readers analysed the samples. In four cases discordant results were obtained. These slides were re-evaluated together and a consensus was reached.

***Positive control.*** The positive control slide was prepared from epidermal tissue, known to contain the antigen. In the positive control tissue all monoclonal antibodies stained similarly.

***Negative control.*** The negative control slide was prepared from the same tissue block as the specimen. Instead of the primary antibody we used a non-immune rabbit serum (Dako code no. X902).

In order to rule out that the difference in staining with the different antibodies is the result of differences in affinity rather than the result of differential expression on tumour cells, we prepared a dilution series probed on positive controls, e.g. skin samples. The dilution factors ranged from 1:50 to 1:400. For CD44v3, CD44v5, CD44v6 and CD44v7–8, optimal staining was found at a dilution factor of 1:100, 1:200, 1:100 and 1:50 of the basic solution (IgG concentration 100 μg $ml^{-1}$) respectively. In order to ensure adequate specificity of the monoclonal antibodies when applied to paraffin-embedded tissue samples, we stained frozen and paraffin-embedded samples of normal vulvar skin side by side with all anti-CD44 variant antibodies. Staining was comparable in all cases.

### Statistics

The chi-square test was used when appropriate. Survival probabilities were calculated by the product limit method of Kaplan and Meier (Kaplan and Meier, 1958). Differences between groups were tested using the log-rank test. The results were analysed for the end point of overall and relapse-free survival. Survival times of patients still alive or relapse-free were censored with the last follow-up date. $P$-values < 0.05 were considered statistically significant. The Kendall Tau b-correlation coefficient was used to assess the correlation between the expression of different CD44 isoforms. The BMDP

© Cancer Research Campaign 1998

**Figure 3** Kaplan–Meier analysis regarding overall survival of patients suffering from tumours with or without expression of splice variant CD44v5

**Figure 4** Kaplan–Meier analysis regarding overall survival of patients suffering from tumours with or without expression of splice variant CD44v7–8

statistical software system (BMDP Statistical Software, Los Angeles, CA, 1990) was used to perform the calculations.

### RESULTS

CD44 isoforms CD44v3, CD44v5, CD44v6 and CD44v7–8 were detected by means of immunohistochemistry in 28% (20/70), 47% (33/70), 33% (23/70) and 17% (12/70) of the tumour samples respectively. We observed a membrane-bound staining pattern in all specimens positive for the investigated CD44 isoforms. All patients showed complete remission after completion of therapy. The range of follow-up was 6.5–86 months (median 56 months). Thirteen patients developed recurrence of disease after 5–73 months (median 35 months). During the observation period, 12 patients died of tumour progression. CD44v6 expression was found in patients with and without recurrence in 14 and 9 cases respectively. Patients suffering from tumours expressing CD44v6 had a poorer relapse-free (log-rank test, $P = 0.02$) and overall survival (log-rank test, $P = 0.03$, Figure 1). CD44v3 expression was found in patients with and without recurrence in 12 and 8 cases respectively. Patients suffering from tumours expressing CD44v3 had a poorer relapse-free (log-rank test, $P = 0.04$) and overall survival (log-rank test, $P = 0.01$, Figure 2).

Survival analysis for the whole population showed no significant prognostic value for CD44v5 (log-rank test, $P = 0.8$, Figure 3) and CD44v7–8 (log-rank test, $P = 0.9$, Figure 4). Correlation coefficients for CD44v3/CD44v5, CD44v3/CD44v6, CD44v3/CD44v7–8, CD44v5/CD44v6, CD44v5/CD44v7–8 and CD44v6/CD44v7–8 were 0.24, 0.01, 0.11, 0.21, 0.12 and – 0.05 respectively.

Patients with poorly differentiated tumours had a shorter overall survival than patients with moderately and highly differentiated tumours (log-rank test, $P = 0.02$). We found no correlation between the expression of CD44 isoforms and histological grade of the tumours.

### DISCUSSION

The expression of cell-surface glycoproteins encoded by CD44 variant exons has been shown to be associated with poor prognosis in human malignancies, e.g. breast, cervical and colorectal cancer (Mulder et al, 1994; Kainz et al, 1995; Kauffmann et al, 1995). We assessed the expression pattern and prognostic impact of CD44 splice variant proteins CD44v3, CD44v5, v6 and v7–8 in 70 primary vulvar carcinomas.

Because of the rarity of the disease, few data on CD44 expression in vulvar cancer have been published so far. The presence of a prominent fibromyxoid stromal response (PFSR) is correlated with clitoral involvement, ulcerative growth and regional lymph node metastasis in vulvar cancer. Ambros et al (1996) have reported PFSR to be strongly correlated with high CD44 expression. Therefore, it may be hypothesized that CD44 plays a role in vulvar tumorigenesis by altering the hyaluronate metabolism of the vulvar stroma.

In our series vulvar cancer samples showed frequent expression of CD44v3, CD44v5 and CD44v6, whereas CD44v7–8 was only expressed in a small number of cases. This expression pattern is in accordance with previously reported data on squamous cell carcinomas of the cervix (Kainz et al, 1995).

In all our specimens we observed a membrane-bound staining pattern for all four investigated CD44 isoforms. This observation is in accordance with previously reported data on CD44 staining patterns in other human malignomas (Salmi et al, 1993; Kainz et al, 1995). The immunohistochemical approach of detecting CD44 expression must be viewed with care because of possible underestimation of membrane molecule expression as a result of embedding procedures. However, in recent studies an excellent correlation between the detection of CD44 isoforms by immunohistochemistry and reverse transcription PCR was reported (Dall et al, 1995).

In two recent pilot studies involving 30 and 25 cases of primary vulvar cancer of different stages, CD44v3 and CD44v6 have been described as new prognostic factors (Tempfer et al, 1996*a, b*). It has to be stated that a total of nine patients described in these previous studies are also incorporated in the present investigation. The aim of the present study was to evaluate whether CD44 isoform expression may be used as an adverse prognosticator in a homogeneous collective of patients with early-stage vulvar cancer. We were able to show that expression of CD44 isoforms containing variant exons v6 or v3 is correlated with a poor prognosis. Patients whose tumours revealed expression of CD44v6 or CD44v3 showed a significantly poorer overall and relapse-free survival.

These data indicate that CD44 isoform expression is an early event in vulvar carcinogenesis. It is a shortcoming of this study that, because of the study period before the introduction of a surgical staging system by FIGO in 1988 (Anonymous, 1989), no groin dissection was performed. Therefore it cannot be ruled out that micrometastases may have been present in patients included in the study. This has to be taken into account when interpreting the results of this study.

The assessment of CD44 isoform expression could be of clinical value in selecting patients for close follow-up schemes or in deciding about adjuvant therapy in patients suffering from low-stage vulvar cancer. In summary, the results of this preliminary study indicate that the evaluation of CD44 expression may be helpful in individualizing the management of early-stage vulvar cancer.

### REFERENCES

Anonymous (1989) FIGO stages – 1988 revision (announcement). *Gynecol Oncol* **35**: 125

Ambros RA, Kallakury B, Malfetano J and Mihm M (1996) Cytokine, cell adhesion receptor, and tumour suppressor gene expression in vulvar squamous carcinoma: correlation with prominent fibromyxoid stromal response. *Int J Gynecol Pathol* **15**: 320–325

Cannistra SA, Abu-Jawdeh G and Niloff J (1995) CD44 variant expression is a common feature of epithelial ovarian cancer: lack of association with standard prognostic factors. *J Clin Oncol* **13**: 1912–1921

Dall P, Heider KH, Sinn HP, Skroch Angel P, Adolf G and Kauffmann M (1995) Comparison of immunohistochemistry and RT-PCR for detection of CD44v-expression, a new prognostic factor in human breast cancer. *Int J Cancer* **60**: 471–477

Hacker NF (1994) Vulvar cancer. In *Practical Gynecologic Oncology*, 2nd edn, Berek JS and Hacker NF (eds), pp. 403–425. Williams and Wilkins: Baltimore

Hermanek P and Sobin L (1992) *UICC TNM Classification of Malignant Tumours*, 4th edn, 2nd rev. Springer-Verlag: Berlin.

Herrlich P, Zoller M, Pals ST and Ponta H (1993) CD44 splice variants: metastases meet lymphocytes. *Immunol Today* **14**: 395–399

Joensuu H, Ristamaki R, Klemi PJ and Jalkanen S (1993) Lymphocyte homing receptor (CD44) expression is associated with poor prognosis in gastrointestinal lymphoma. *Br J Cancer* **68**: 428–432

Kainz C, Kohlberger P, Sliutz G, Tempfer C, Heinzl H and Reinthaller A (1995) Splice variants of CD44 in human cervical cancer stage IB to IIB. *Gynecol Oncol* **57**: 383–387

Kaplan EL and Meier P (1958) Nonparametric estimation from incomplete observations. *J Am Stat Assoc* **53**: 457–481

Kaufmann M, Heider KH, Sinn HP, von Minckwitz G, Ponta H and Herrlich P (1995) CD44 variant exon epitopes in primary breast cancer and length of survival. *Lancet* **345**: 615–619

Kucera H and Weghaupt K (1988) The elecrosurgical operation of vulvar carcinoma with post-operative irradiation of inguinal lymph nodes. *Gynecol Oncol* **29**: 158–167

Mackay CR, Terpe H, Stauder R, Marston W, Stark H and Guenthert U (1994) Expression and modulation of CD44 variant isoforms in humans. *J Cell Biol* **124**: 71–82

Mulder JW, Kruyt PM, Sewnath M, Oosting J, Seldenrijk CA and Weidema WF (1994) Colorectal cancer prognosis and expression of exon-v6-containing CD44 proteins. *Lancet* **344**: 1470–1472

Salles G, Zain M, Jiang W, Boussiotis V and Shipp M (1993) Alternatively spliced CD44 transcripts in diffuse large cell lymphomas: characterization and comparison with normal activated B cells and epithelial malignancies. *Blood* **82**: 3539–3547

Salmi M, Gron Virta K, Sointu P, Greman R, Kalimo H and Jalkanen S (1993) Regulated expression of exon v6 containing isoforms of CD44 in man: downregulation during malignant transformation of tumours of squamocellular origin. *J Cell Biol* **122**: 431–442

Screaton GR, Bell MV, Jackson DG, Cornelis FB, Gerth U and Bell JI (1992) Genomic structure of DNA encoding the lymphocyte homing receptor CD44 reveals at least 12 alternatively spliced exons. *Proc Natl Acad Sci USA* **89**: 12160–12164

Smith CW, Patton JG and Nadal Ginard B (1989) Alternative splicing in the control of gene expression. *Annu Rev Genet* **23**: 527–577

Tempfer C, Gitsch G, Haeusler G, Reinthaller A, Koelbl H and Kainz C (1996*a*) Prognostic value of immunohistochemically detected CD44 expression in patients with carcinoma of the vulva. *Cancer* **78**: 273–277

Tempfer C, Gitsch G, Hanzal E, Reinthaller A, Koelbl H and Kainz C (1996*b*) Expression of the adhesion molecule CD44v3 is a prognostic factor in vulvar carcinoma. *Anticancer Res* **16**: 2029–2032

Underhill C (1992) CD44: the hyaluronan receptor. *J Cell Sci* **103**: 293–298

© Cancer Research Campaign 1998